# Generalized pollination of morphologically specialized *Vincetoxicum hirundinaria* (Apocynaceae, Asclepiadoideae)

**DOI:** 10.1093/aobpla/plag048

**Published:** 2026-10-05

**Authors:** Wujian Xiong, Jeff Ollerton, Sigrid Liede-Schumann, Ulrich Meve, Herbert C Wagner, Wenhui You, Junpeng Mu, Stefan Dötterl

**Affiliations:** School of Life Sciences (School of Ecological Forestry), Mianyang Teachers’ College, Mianyang 621000, China; Department of Environment and Biodiversity, University of Salzburg, Salzburg 5020, Austria; School of Ecological and Environmental Sciences, East China Normal University, Shanghai 200241, China; Faculty of Arts, Science and Technology, University of Northampton, Waterside Campus, Northampton NN1 5PH, United Kingdom; Key Laboratory for Plant Diversity and Biogeography of East Asia, Kunming Institute of Botany, Chinese Academy of Sciences, Kunming 650201, China; Department of Plant Systematics, University of Bayreuth, Bayreuth 95440, Germany; Department of Plant Systematics, University of Bayreuth, Bayreuth 95440, Germany; Institute of Biology, University of Graz, Universitätsplatz 2/I, Graz 8010, Austria; School of Ecological and Environmental Sciences, East China Normal University, Shanghai 200241, China; School of Life Sciences (School of Ecological Forestry), Mianyang Teachers’ College, Mianyang 621000, China; Department of Environment and Biodiversity, University of Salzburg, Salzburg 5020, Austria; Centre for Climate Change Resilience (CC:R), University of Salzburg, Salzburg 5020, Austria

**Keywords:** central Europe, floral morphology, generalist pollinators, pollen transfer efficiency, pollinarium, pollination biology

## Abstract

Generalized pollination systems with ecologically and/or functionally diverse pollinator assemblages are common in nature. However, the extent to which a plant with specialized floral morphology can exhibit a generalized pollination system remains poorly understood. We investigated this question using *Vincetoxicum hirundinaria* Medik. (Apocynaceae, Asclepiadoideae) as a model system—a species whose morphologically specialized flowers are described as being visited by flies, bees, and moths. In preliminary observations, we even found various other insects as flower visitors. We hypothesized that *V. hirundinaria* has a more diverse pollinator spectrum than previously known. To test this hypothesis, we studied the flower biology and pollination ecology of *V. hirundinaria* at several sites in and around Salzburg. Our findings reveal that the self-incompatible, pale white to ivory-coloured flowers open between 4 p.m. and 6 p.m., and attract a diverse community of both diurnal and nocturnal pollinators, including cockroaches, beetles, and ants not previously reported as pollinators of *V. hirundinaria*. Pollen transfer efficiency and fruit set were low. A high number of non-pollinator species got trapped by the flowers, often fatally. Overall, *V. hirundinaria* has a more generalized pollination system than previously recognized, showing that a complex floral morphology can coexist with ecological and functional generalization.

## Teaser

Many plants have flowers specialized for particular pollinators, but we show that *Vincetoxicum hirundinaria* attracts a surprisingly broad range of insects, including day-flying and night-flying species, and previously unknown pollinators, such as cockroaches, beetles, and ants. Its flowers open in the late afternoon, pollen transfer and fruit production are low, and many non-pollinating insects are trapped and killed. This species therefore shows that complex floral structures can coexist with a generalized pollination system.

## Introduction

Flowering plants exhibit a remarkable diversity of pollination systems, ranging from highly specialized interactions with a single pollinator species to generalized strategies involving multiple pollinator groups (Johnson and Steiner 2000, Ollerton et al. 2007, Armbruster 2017). While specialized systems have long captivated researchers (e.g. due to their co-evolutionary dynamics), generalized pollination systems remain less well understood. This is particularly true of those species that are ecologically and functionally generalized but have specialized floral structures. Here, we use ecological generalization to refer to visitation by a taxonomically diverse assemblage of animals, functional generalization to effective pollen transfer by multiple functionally distinct pollinator groups, and phenotypic specialization to floral traits that constrain visitor access or precisely control pollen removal and deposition (see Ollerton et al. 2007). Pollination systems that possess ecological and functional generalization but are phenotypically specialized are especially intriguing as they raise fundamental questions about how different pollinators functionally interact with the specialized flowers, and how these interactions influence reproductive success of the plants (Fenster et al. 2004, Schiestl and Johnson 2013).

The distinction between specialization and generalization in pollination systems is conceptually complex because plants may be specialized in some respects but generalized in others. A species can possess highly specialized floral morphology, restrict access to rewards, or precisely place pollen on visitors, while still being visited and effectively pollinated by a taxonomically or functionally diverse assemblage of animals. Conversely, apparently open flowers may depend reproductively on only a small subset of visitors. Specialization is expected to evolve where particular pollinators provide more reliable, efficient, or high-quality pollen transfer, whereas generalization may be favoured where pollinator availability is spatially or temporally variable, where several visitor groups provide comparable fitness returns, or where attracting many potential pollinators buffers plants against pollinator limitation (Waser et al. 1996, Ollerton et al. 2007, Armbruster 2017). Floral anthesis and longevity are also relevant to this question because they define the temporal window over which different visitor groups can interact with flowers: short, time-restricted anthesis may indicate specialization on pollinators active during a narrow period, whereas dusk anthesis combined with multi-day floral longevity may increase opportunities for both diurnal and nocturnal insects to act as pollinators.

This distinction is especially relevant in Asclepiadoideae, where the highly derived pollinarium mechanism imposes a strong morphological filter on visitors. During a visit, an insect appendage may enter a guide rail and engage with the corpusculum; as the insect withdraws, it can remove the attached pollinarium. On a subsequent visit, a pollinium may be guided into a stigmatic chamber. Because successful removal and deposition depend on the size, shape, and strength of the visitor, this mechanism acts as a mechanical filter of potential pollinators. Nevertheless, pollination systems in this group range from extreme specialization to broad generalization (Ollerton and Liede 1997, Endress et al. 2014, Endress 2015, Ollerton et al. 2019). A large genus within Asclepiadoideae is *Vincetoxicum* Wolf, which comprises more than 250 species (Endress et al. 2018, Shah et al. 2021, POWO, assessed on 10 August 2025). Despite its substantial diversity, the ecological and reproductive biology of most *Vincetoxicum* species remains poorly explored. In the past, four insect orders, all visiting the flowers for their nectar, have mostly been reported as diurnal and/or nocturnal pollinators, i.e. Diptera, Lepidoptera, Coleoptera, and Hymenoptera (Lumer and Yost 1995, Yamashiro et al. 2008, Mochizuki 2025). Additionally, nocturnal Blattodea (cockroaches) were recently added to the list of effective pollinators in *Vincetoxicum* (Xiong et al. 2020).

*Vincetoxicum hirundinaria* Medik., one of the few Central European representatives of the Apocynaceae, is a useful system for testing the strength and limits of specialization: Its flowers have the complex pollinarium, gynostegium, and corona typical of milkweeds. The pollinarium is composed of two pollinia and a corpusculum. The gynostegium is formed by the post-genital fusion of stamens and carpels, with the narrow guide rails formed between adjacent anthers that direct insect appendages towards the corpusculum and stigmatic chamber. Despite this complex morphology, previous observations suggest that different populations may be pollinated by flies, bees, or moths (Timonin and Savitsky 1997, Leimu 2004); in preliminary observations we also found other potential pollinators visiting the flowers, such as beetles and ants. Quantifying both the full visitor spectrum and the relative effectiveness of different visitor groups allows us to ask whether this species is genuinely generalized, or whether apparent generalization masks functional dependence on a narrower subset of effective pollinators.

Because the species is reported to be predominantly outcrossing and self-incompatible, but with some population-level variation in mating-system expression (Leimu 2004), we first assessed the breeding system of some populations in Salzburg to establish their dependence on pollinator-mediated pollen transfer. We then tested three predictions arising from the specialization–generalization framework. First, if *V. hirundinaria* is ecologically generalized, its flowers should attract a taxonomically diverse assemblage of visitors. Because the composition and availability of potential pollinators can vary spatially, we additionally tested whether the visitor assemblage differed among sites. Second, if ecological generalization translates into functional generalization, pollinaria should be removed and carried by multiple visitor groups rather than by a single pollinator taxon. Third, if the specialized floral mechanism imposes a stronger functional filter than visitor diversity alone suggests, effective pollination should be concentrated in a narrower subset of visitors. We therefore asked whether *V. hirundinaria* combines morphological specialization with ecological and functional generalization, or whether apparent generalization masks dependence on a smaller set of effective pollinators.

## Materials and methods

### Study site and species

*Vincetoxicum hirundinaria* Medik. is a herbaceous perennial with a wide geographical distribution, native to most parts of Europe, Algeria and Morocco, Belarus, Ukraine, Western Siberia, and introduced to some parts of North America. This study was conducted from April to October 2021 in and around Salzburg at five sites, including one site in the Botanical Garden of the University of Salzburg, where a population of this species spontaneously became established (Supplementary Table S1.1). In Salzburg, *V. hirundinaria* has a patchy distribution along the mountain slopes, with individuals particularly abundant at the top of Mount Gaisberg. We typically found it on slopes and rocky areas, often adjacent to forest roads. Approximately 40 to more than 300 flowering individuals were found at the different sites (Supplementary Figure S1.1).

### Phenology and floral morphology

The flowering and fruiting seasons were recorded in 2021. As the floral structures are highly specialized (e.g. gynostegium, guide rail, pollinaria, and style-head) (Nam and Chung 2018) (Supplementary Figure S1.2), three individual plants were randomly selected per site to assess these floral traits. For each individual, one randomly selected fully open flower was examined, and the following traits were recorded under a microscope (S8 APO, Leica, Germany): corolla diameter (CLD), corona diameter (CND), length of corona lobe (CNL), width of corona lobe (CNW), length of gynostegium (GYL), diameter of the gynostegium (GYD), length of the guide rail (GRL), width of the guide rail (GRW), length of the stamen to stigmatic chamber (SSL), length of the corpusculum (COL), width of the corpusculum (COW), length of the pollinia (POL), and width of the pollinia (POW). To obtain a flower average of CNL, CNW, GRL, GRW, SSL, COL, COW, POL, and POW, two of the five available structures were randomly selected and measured.

To determine the anthesis of the flowers, three individual plants were randomly selected per site. For each individual, 15 flower buds (five per inflorescence) from three inflorescences were randomly selected and labelled, and their opening status was recorded hourly over 24 hours. To assess the longevity of individual flowers and inflorescences, three individual plants were randomly selected per site. From each, two flower buds and two inflorescences were labelled and observed daily to record their lifespan.

### Breeding system

To determine the breeding system of *Vincetoxicum hirundinaria*, we transplanted five seedlings each from three (to reduce the likelihood of relatedness) of the sites (Obergnigl, Kapuzinerberg, Kapuzinerkloster) to the Botanical Garden of the University of Salzburg. Seedlings were planted outdoors in pots filled with a mixture of sand and soil, which facilitated watering while maintaining growth environment as natural as possible. During the peak flowering in 2021, four pollination treatments were conducted (Supplementary Table S1.2): (i) Spontaneous selfing: Five flower buds per individual were enclosed in fine mesh nylon bags (12 cm × 18 cm) to exclude pollinators. (ii) Open-pollination: Ten flower buds per individual were left unmanipulated but labelled to track development from anthesis to fruit maturation. (iii) Hand self-pollination: Five bagged flower buds per individual were manually self-pollinated by extracting pollinaria and inserting one pollinium into the stigmatic chamber of the same flower with a stainless steel needle (145 mm in length, 0.05 mm tip diameter) under a dissecting microscope. (iv) Hand cross-pollination: Ten bagged flower buds per individual were cross-pollinated using pollinia from a single donor. Each individual was used as a pollen donor only once, and the donors and recipients were randomized. For all treatments, we recorded the number of pods initiated, matured, and aborted. Fruit set and abortion rates were calculated as: Initial fruit set rate = (The number of pods initiated/The number of flowers treated) × 100%. Abortion rate = (The number of pods aborted/The number of pods initiated) × 100%.

### Flower visitors and pollinators

During the peak flowering season in mid-June, visiting insects were observed in the field during both the day and the night under rainless weather conditions (Mochizuki et al. 2017). For daytime observations, one plant individual was monitored from a distance of 0.8 m at a time for 10 minutes, with six individuals observed in total of 60 min (treated as a session). All insects observed on these six plants during the 60 minutes were pooled to build a replicate (session). Per site, 3–11 daytime sessions were performed on different plant individuals (18–66 plants observed at each site). Nighttime observations involved monitoring one plant individual at a time for 30 minutes under red flashlight to minimize the potential impact of light on flower visitors, with the observations of two plant individuals pooled to a session. Per site, 2–10 nighttime sessions were performed on different plant individuals (4–20 plants observed at each site). In total, 30 hours of daytime and 20 hours of nighttime observations were performed. During these observations, the number of floral visitors and the frequency and duration of visits were recorded. For each visitor species/morphotype, at least one individual was captured for further identification to the lowest possible taxonomic level and to check for the presence of pollinaria (with or without attached pollinia) under a microscope. Visitors with pollinaria attached to their bodies were considered as pollinators of *V. hirundinaria*. In the study area, no other Asclepiadoideae species occur, thus, pollinaria can only come from the study plant species. Voucher specimens of all visiting insects were deposited at the Plant Ecology Laboratory of the University of Salzburg.

### Pollinaria carrying rate

To determine the pollinaria carrying rate, 5–200 further individuals of each taxon of pollinator, depending on availability, were randomly captured after flower visits, using a net or a plastic bottle fitted with a sponge, and the number and percentage of individuals with pollinaria attached was determined (Wiemer et al. 2012). The mean number of pollinaria carried per individual was also determined (considering only individuals with at least one pollinarium).

### Pollination rate

To explore the pollination success of pollinator groups at different periods of the day and night, 30–60 plant individuals were randomly selected per site. For each individual, 10 flower buds (five per inflorescence) from two inflorescences were tagged and enclosed in a single bag. When they all opened, two treatments were applied: One group of bags (*n* = 20–40) was removed at sunrise, and flowers (*n* = 200–400) were exposed to daytime visitor insects until sunset, while another group of bags (*n* = 10–20) was removed at sunset, and flowers (*n* = 100–200) were exposed to nighttime visitor insects until sunrise the following day.

For each tagged flower, pollinaria removal and pollinia insertion were recorded under a 20× hand lens. The pollinaria removal rate was calculated by dividing the number of flowers with at least one pollinarium removed by the number of flowers examined, and multiplying the obtained value by 100%. The pollinia insertion rate was calculated by dividing the number of flowers with at least one pollinium inserted by the number of flowers examined, and multiplying the obtained value by 100%. For each selected individual, the pollen transfer efficiency (PTE) was calculated as: (the numbers of pollinia inserted)/2 × (the numbers of pollinaria removed) × 100%.

### Statistical analyses

A Kruskal–Wallis analysis followed by a Dunn’s *post hoc* test was used in Statistica 14.0 (Cloud Software Group Inc. 2023) to test for differences in the duration of a flower visit among insect orders, treating bees and ants within the Hymenoptera separately due to their different behaviours. Similarly, differences of pollinaria carrying among different pollinator groups and pollination rate among sites at different periods of the day and night were evaluated using a Kruskal–Wallis analysis followed by Dunn’s *post hoc* test. To test for differences in relative visitor communities (contribution of single taxa to the overall recorded visitor individuals) at the insect order level (with treating ants and bees within the Hymenoptera again separately) among the study sites and between daytime and nighttime, two-factorial PERMANOVA analyses were performed in Primer 7 (Clarke and Gorley 2015). This analysis was conducted on pairwise Bray–Curtis similarities of square-root transformed relative visitor assemblages calculated among all pairs of observation sessions (fixed factors in crossed-design: site, time of observation; 10 000 permutations). To visually represent similarities in floral visitors among sites and times, non-metric multi-dimensional scaling based on the Bray–Curtis similarities was employed. To evaluate how well the configuration produced the similarity matrix the stress value is given. The smaller the stress value, the better is the fit of the reproduced ordination to the observed distance matrix (Clarke and Gorley 2015).

## Results

### Phenology and floral morphology

At all sites except Gaisberg, flowering started in the middle of May and continued until the end of July. Fruits generally ripened in late July or early August. At Gaisberg, the phenology was delayed by 1 month.

Each inflorescence consisted of 5–26 flowers (median = 12, *n* = 100), with 1.4 ± 0.6 (mean ± SD, *n* = 70) flowers simultaneously open. The flowers opened daily between 16:00 and 18:00, taking 15–20 minutes to fully open, without closing again. The lifespan of a flower was 6.2 ± 1.3 (mean ± SD, *n* = 30) days, while an inflorescence flowered for 25.8 ± 3.4 (mean ± SD, *n* = 30) days. Nectar accumulated in the stigmatic chambers and the flowers emitted a heavy, sweet scent during day and night.

The flowers were 12.0 mm ± 1.2 mm (mean ± SD, *n* = 15) in diameter, and had a five-lobed corolla accompanied by 5 calyx glands (Supplementary Figure S1.3). The pale green corona measured 2.25 ± 0.11 mm (mean ± SD, *n* = 15) in diameter. The gynostegium had a diameter of 0.86 ± 0.05 mm (mean ± SD, *n* = 15), and the guide rail measured 0.60 ± 0.04 mm (mean ± SD, *n* = 15) in length. The pollinarium consisted of a brown corpusculum and two oblong pollinia. The corpusculum measured 200 µm (± 20 µm) × 90 µm ± 10 µm and a pollinium measured 230 µm (± 20 µm) × 70 µm (± 20 µm) (mean ± SD, *n* = 15) (Supplementary Figure S1.2).

### Breeding system

Hand pollination experiments revealed that neither bagged nor self-pollinated flowers of *Vincetoxicum hirundinaria* produced fruit (*n* = 75), indicating that *V. hirundinaria* possesses no ability for spontaneous selfing and is self-incompatible.

The initial fruit set rates of open pollination and cross-pollination treatments were 3.3% (*n* = 150) and 25.3% (*n* = 150), respectively. The fruit abortion rates were 40.0% (*n* = 5) for open pollination and 42.0% (*n* = 38) for cross-pollination. The mature fruit set was 2.0% (*n* = 150) for open pollination and 14.0% (*n* = 150) for cross-pollination.

### Flower visitors and pollinators

During the 50 hours of observations, a total of 2289 daytime and 642 nighttime insect visits to flowers were recorded. The main flower visitors belonged to seven insect orders (Fig. 1a): Blattodea, Coleoptera, Diptera, Hemiptera, Hymenoptera, Lepidoptera, and Mecoptera. Overall, the flower visitors represented 38 families and 81 species. Diptera [40 (morpho)species] and Lepidoptera (14 species) were most diverse, with lower numbers of species (1–8) in the other orders (Fig. 2, Supplementary Table S2.1).

**Figure 1:**
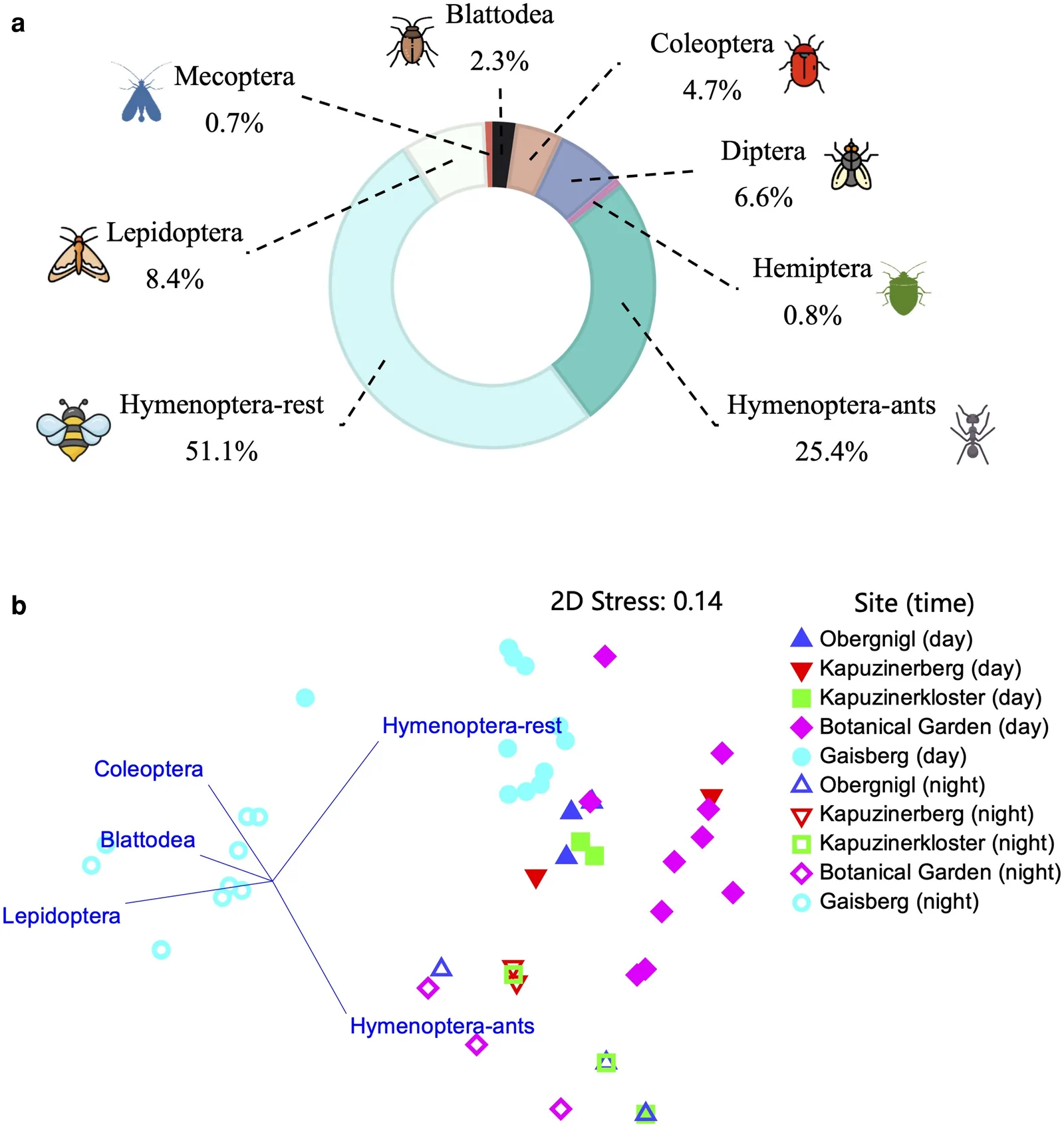
(a) Proportion of visits (%) of the seven insect orders to flowers of *Vincetoxicum hirundinaria*. Insect icons are licensed under https://www.flaticon.com/. (b) Non-metric multidimensional scaling used to display (dis)similarities in relative flower visitor communities of *Vincetoxicum hirundinaria* at the insect order level (ants and bees within the Hymenoptera were treated separately) among the observation session performed at different sites and during daytime (filled symbols) and at night (open symbols). The insect orders most responsible for the ordination are also given.

**Figure 2:**
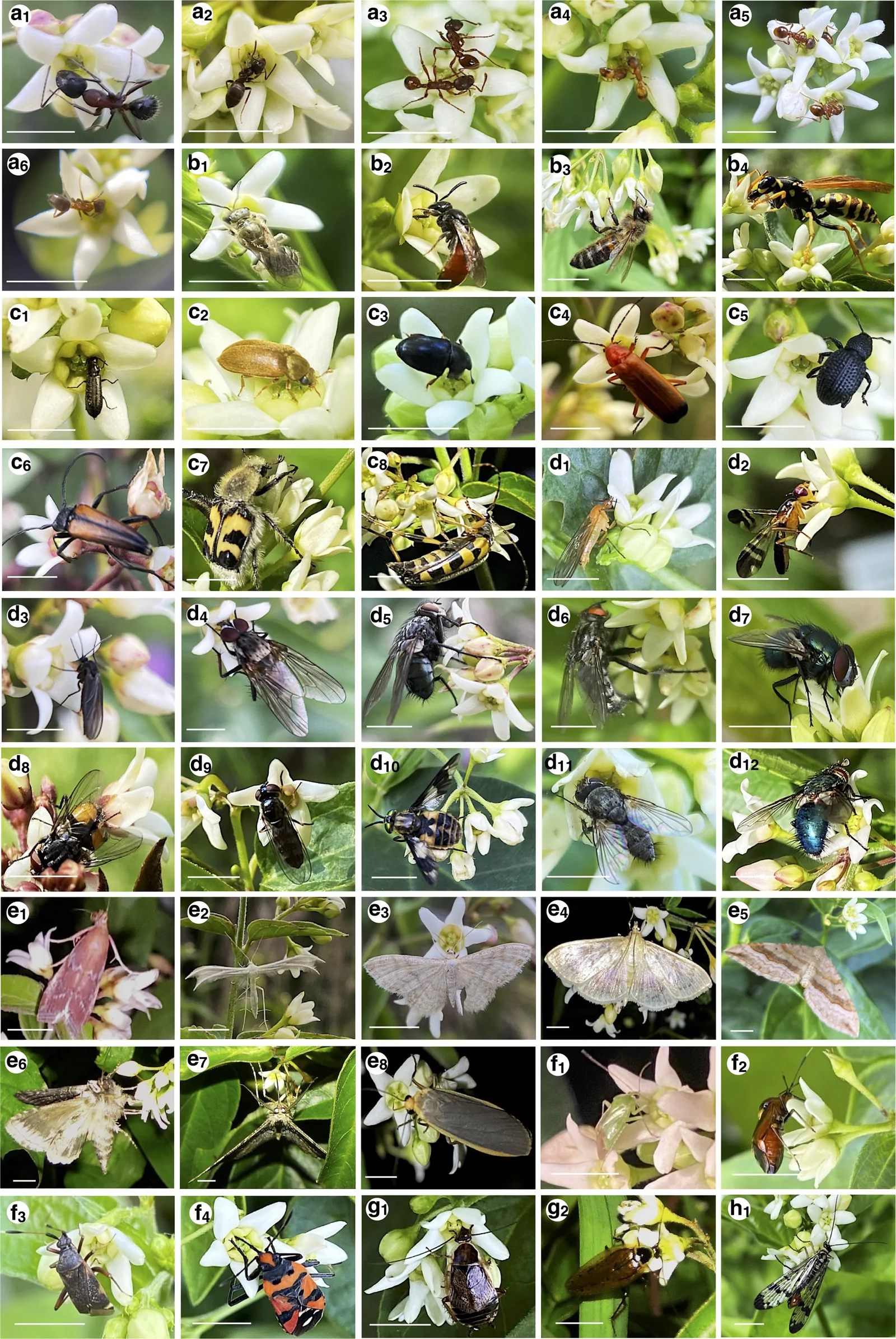
Examples of flower visitors of Vincetoxicum hirundinaria. Scale bar = 5 mm. (*a*1) *Camponotus ligniperda/herculeanus*. (*a*2) *Tapinoma erraticum/subboreale*. (*a*3) *Myrmica rubra/ruginodis*. (*a*4) *Temnothorax unifasciatus* group. (*a*5) *Myrmica* sp. (*a*6) *Lasius emarginatus*. (*b*1) *Lasioglossum* sp. (*b*2) *Sphecodes* sp. (*b*3) *Apis mellifera*. (*b*4) *Polistes dominula*. (*c*1) *Dasytes* sp. (*c*2) *Byturus* sp. (*c*3) *Meligetinae* sp. (*c*4) *Rhagonycha fulva*. (*c*5) *Otiorhynchus* sp. (*c*6) *Stenurella melanura*. (*c*7) *Trichius fasciatus*. (*c*8) *Rutpela maculata*. (*d*1) *Lauxaniidae* sp. (*d*2) *Tephritidae* sp. (*d*3) *Sciaridae* sp. (*d*4) *Diptera* sp. (*d*5) *Calliphoridae* sp. 1. (*d*6) *Sarcophagidae* sp. (*d*7) *Calliphoridae* sp. 2. (*d*8) *Muscidae* sp. (*d*9) *Melanostoma* sp. (*d*10) *Diptera* sp. (*d*11) *Diptera* sp. (*d*12) *Calliphoridae* sp. 3. (*e*1) *Pyralidae* sp. (*e*2) *Pterophoridae* sp. (*e*3) *Idaea dilutaria*. (*e*4) *Patania ruralis*. (*e*5) *Scotopteryx chenopodiata*. (*e*6) *Autographa gamma*. (*e*7) *Alcis deversata*. (*e*8) *Eilema depressa*. (*f*1) *Miridae* sp. (*f*2) *Deraeocoris ruber*. (*f*3) *Capsodes* sp. (*f*4) *Lygaeus equestris*. (*g*1) *Ectobiidae* sp., nymph. (*g*2) *Ectobiidae* sp., adult. (*h*1) *Panorpidae* sp. Different uppercase letters represent different insect orders, with ants, bees, and wasps treated separately.

Hymenoptera accounted overall for 76.5% of the visits (51.1% were by bees and wasps, and 25.4% by ants), while the remaining orders contributed between 0.7% and 8.4% of the visits (Fig. 1a).

During daytime, Coleoptera were more abundant at Gaisberg than at the other sites, which were more strongly dominated by Hymenoptera (Fig. 1b, Supplementary Figure S2.1). At night, plants at Gaisberg were mainly visited by Lepidoptera, followed by Blattodea and Coleoptera, with visitor communities of plants of the other sites, including the Botanical Garden, strongly dominated by ants (Fig. 1b, Supplementary Figure S2.2). Within the Hymenoptera, ants were recorded as flower visitors both day and night, with bees recorded only during the day.

At the insect order level, but with treating ants and bees within the Hymenoptera separately, visitor assemblages differed among study sites (PERMANOVA: pseudo-*F*_4,40_ = 11.38, *P* < .001) and between day- and nighttime observations (PERMANOVA: pseudo-*F*_1,40_ = 25.21, *P* < .001), with a significant interaction effect (PERMANOVA: pseudo-*F*_4,40_ = 6.99, *P* < .001). Post-hoc analyses revealed that during both day and night, Gaisberg plants had a different visitor assemblage than plants on the other sites (*P* < .042), with no differences among plants of the other sites (*P* > .099). In Gaisberg and the Botanical Garden (*P* < .003), but not at the other sites (*P* > .099) where samples sizes were quite small, assemblages differed between daytime and nighttime.

The analysis of median flower visit duration revealed significant differences across insect orders (Fig. 3a; Kruskal–Wallis test: *H* (7, *n* = 1690) = 430.99, *P* < .001). With 2 s in the median, flower visits of Diptera were shorter than the visits of any other order (Fig. 3a). Hymenoptera (bees and wasps) showed the second lowest median visit durations (16 s), shorter than visits of any insect order with longer durations, except Mecoptera (Fig. 3a). Median flower visit durations of insects other than Diptera and Hymenoptera (bees and wasps) were between 25 and 50 s (Fig. 3a).

**Figure 3:**
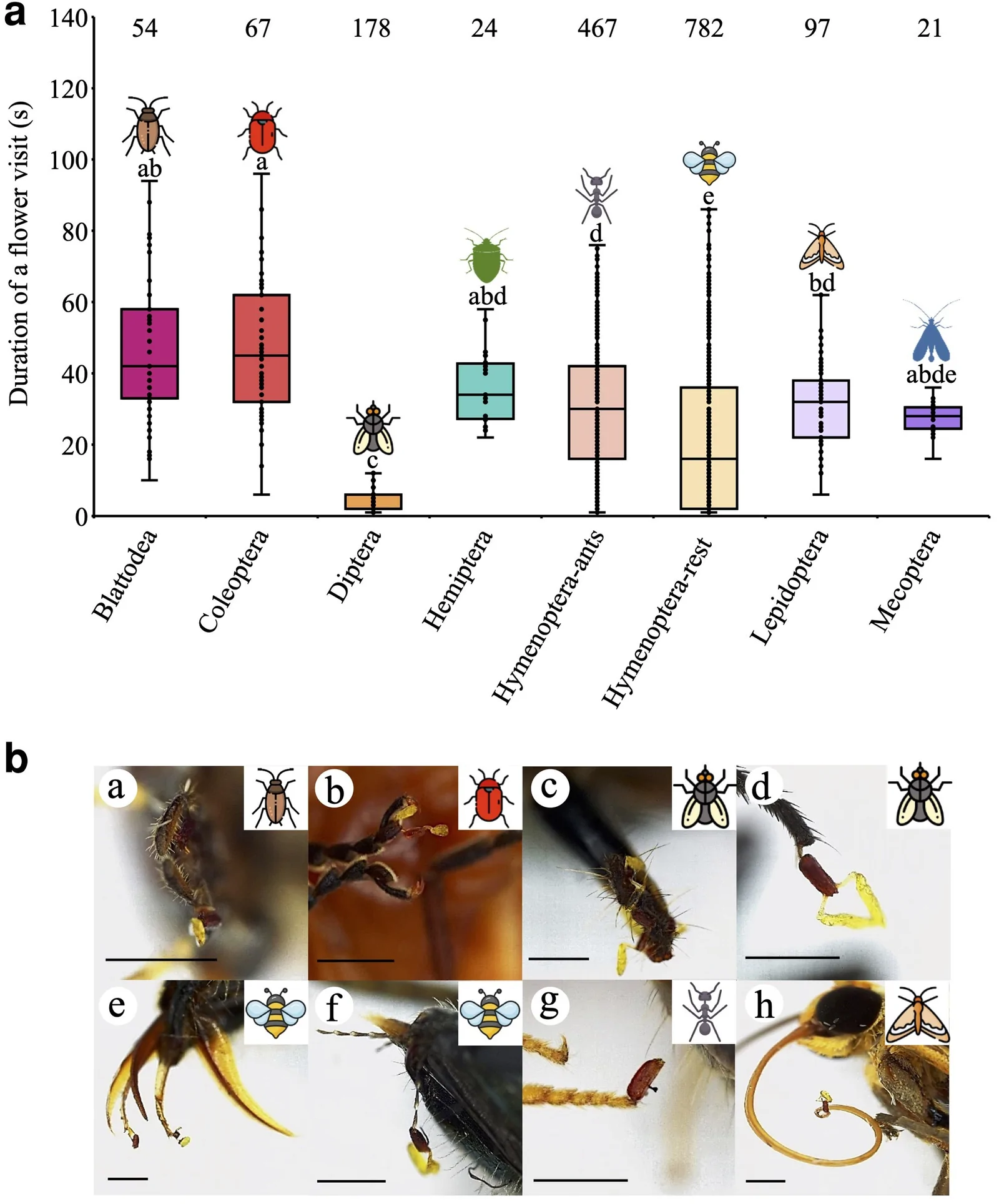
(a) Duration of visits by insects of different orders (bees and wasps, and ants within Hymenoptera treated separately) to flowers of *Vincetoxicum hirundinaria*. Sample sizes are shown above the boxplots. (b) Pollinators of *Vincetoxicum hirundinaria* with pollinaria attached to their body parts. Scale bar = 1 mm. (a) *Ectobiidae* sp. cockroach with pollinaria on labial palps. (b) *Rhagonycha fulva* beetle with pollinaria on its legs. (c) *Calliphoridae* sp. fly with pollinaria on its proboscis. (d) *Anthomyiidae* sp. fly with a pollinarium on a leg. (e) *Apis mellifera* bee with pollinaria on its mouthparts. (f) *Lasioglossum pauxillum* bee with a pollinarium on its labial palps. (g) *Camponotus herculeanus* ant with a pollinarium on a leg. (h) *Eilema depressa* moth with a pollinarium on its proboscis.

Additionally, we observed that individuals from 16 morphospecies of flies from eight families were captured by the flowers and died, mostly with the probiscis seized in a corpusculum (Supplementary Table S3.1, Supplementary Figure S3.1).

Among all the flower visitors, we identified a diverse assemblage of pollinators. In total, 5 orders, 18 families and 37 (morpho)species carried pollinaria and were identified as pollinators: 15 (morpho)species from 7 families of flies (Diptera), 7 species from 4 families each of wasps/bees (Hymenoptera) and moths (Lepidoptera), 6 species from ants (Hymenoptera), and 1 species each from cockroaches (Blattodea) and beetles (Supplementary Table S3.2). The pollinaria were attached to the proboscis and leg segments of flies, the proboscis and labial palps of bees/wasps, the proboscis of moths, leg segments of ants and beetles, and labial palps of cockroaches (Fig. 3b).

### Pollinaria carrying rate

Beetles and moths each had a pollinaria carrying rate of 60.0% (*n* = 10 for beetles, *n* = 20 for moths), bees and wasps of 50.0% (*n* = 22), flies of 54.8% (*n* = 42), cockroaches of 41.7% (*n* = 12), and ants of 3.5% (*n* = 200) (Fig. 4a).

**Figure 4:**
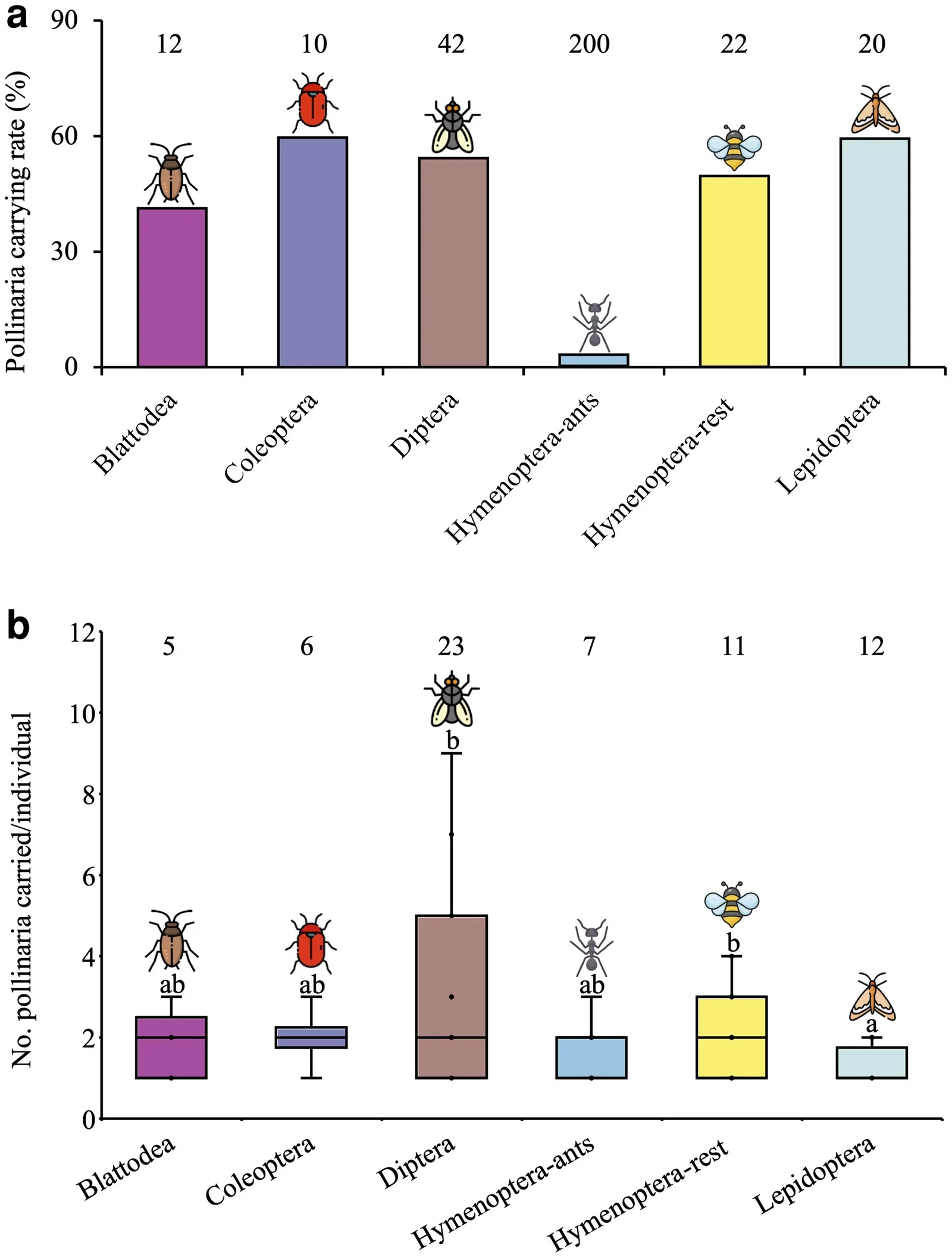
Pollen export of pollinator insect groups. (a) Pollinaria carrying rate. (b) Number of pollinaria carried per individual. Sample sizes are shown above the boxplots.

The analysis of the median number of pollinaria carried revealed significant differences across insect orders (Fig. 4b) (Kruskal–Wallis test: *H* (5, *n* = 64) = 31.27, *P* < .001). Lepidoptera carried in the median only one pollinarium, less than Diptera and Hymenoptera (bees and wasps) that carried in the median two pollinaria each. The other insects carried average pollinaria numbers (Fig. 4b).

### Pollination rate

In all sites except Gaisberg, pollinaria removals only occurred during daytime (Supplementary Figure S4.1). The pollinaria removal rate was similar and on average between 20% and 30% among the different sites during the day, whereas at Gaisberg, the nocturnal removal rate averaged 44% (Kruskal–Wallis test: *H* (*5*, *n* = 168) = 47.86, *P* < .001).

Similarly, in all sites except Gaisberg, pollinaria insertions only occurred during the day (Supplementary Figure S4.1). The pollinia insertion rates were similar across sites during the day, averaging between 2% and 5%, whereas at Gaisberg, the nocturnal insertion rate averaged 12% (Kruskal–Wallis test: *H* (5, *n* = 168) = 28.45, *P* < .001).

Pollen transfer efficiency (PTE) per individual during the day was 5% (*n* = 40) at Obergnigl, 7% (*n* = 30) at Kapuzinerkloster, 4% (*n* = 26) at Kapuzinerberg, 9% (*n* = 20) at Gaisberg, and 3% (*n* = 32) at the Botanical Garden. At night, it was on average 11% (*n* = 20) at Gaisberg (Supplementary Table S4.1).

## Discussion

In this study we investigated the floral and pollination biology of *Vincetoxicum hirundinaria* and found that it is ecologically and functionally generalized. At our study sites, *V. hirundinaria* has highly diverse visitor and pollinator spectra, the visitor and pollinator composition varies among sites, and nocturnal pollination was particularly effective at Gaisberg. Specifically, the flowers of *V. hirundinaria* open at dusk, produce nectar, and emit a strong and sweet odour reminiscent of red wine, both during the day and at night. Flowers are visited and pollinated by a diverse array of insects during both periods. Hymenoptera (mainly bees and ants) were by far the most abundant visitors, followed by Lepidoptera, Diptera, and Coleoptera. Between 40% and 60% of the individuals of the different insect groups carried pollinaria of the plant, with the exception of the ants, which only rarely carried pollinaria. In most study sites, pollinaria were only removed and deposited during the day, except in one site, Gaisberg, where moths were abundant visitors. Altogether, our study expands the known pollinator list of *V. hirundinaria* to include cockroaches, beetles, and ants, demonstrating that this species has a more generalized pollination system than previously recognized (Timonin and Savitsky 1997, Leimu 2004), with significant spatial variation in the identities of the effective pollinators.

### Floral anthesis and longevity

Floral anthesis and longevity are important components of pollination strategy because they determine when flowers are available to different visitor groups and how long individual flowers remain receptive to effective pollen transfer (van Doorn and van Meeteren 2003, van Doorn and Kamdee 2014). In *V. hirundinaria*, flowers opened at dusk and remained open for several days, a pattern that may increase exposure to both late-active diurnal insects and nocturnal visitors. Dusk anthesis may be favoured where nocturnal or crepuscular pollinators provide efficient pollen transfer, as shown in other systems with mixed day- and night-active pollinator assemblages (Miyake and Yahara 1998, 1999; Young 2002). However, anthesis late in the day may also reduce exposure to daytime heat, ultraviolet radiation and water stress, particularly in open or rocky habitats. Multi-day floral longevity may further buffer plants against temporal unpredictability in pollinator activity, acting as a form of bet-hedging when effective visits are infrequent, spatially variable, or concentrated during particular periods of the day or night. In our study, nocturnal pollination was effective mainly at Gaisberg, where moths and cockroaches were recorded, whereas daytime visitors dominated pollination at the other sites. This pattern is consistent with previous studies showing that nocturnal pollinators can sometimes be more effective than diurnal visitors, even when they are less abundant (Fleming et al. 1996, Young 2002, Vanderplanck et al. 2020). Thus, dusk anthesis and extended floral longevity do not indicate specialization on nocturnal pollinators alone; rather, they are consistent with a strategy that maintains access to a broad temporal window of potential pollinators while allowing local visitor assemblages to determine which groups contribute most to pollen transfer.

### Visitors and pollinators

Through systematic field observations and experimental manipulations, we conducted a comprehensive survey of the pollination ecology of *V. hirundinaria*, documenting all flower visitors and confirming their roles in removing pollinaria from the flowers. Similar to the results obtained in Finland (Leimu 2004), we also identified bees as abundant diurnal flower visitors at all our study sites, moths, however, were abundant nocturnal visitors only at the Gaisberg site. Diptera were recorded in all study sites, but contributed only a minor proportion of visits. Overall, data available on flower visitors of *V. hirundinaria* show that visitor communities vary among populations, while sites differ strongly in environmental parameters such as elevation and temperature. We observed a highly diverse assemblage of floral visitors, with a much higher number of pollinator species than previously documented (Timonin and Savitsky 1997, Leimu 2004). These include cockroaches (Ectobiidae sp., both nymphs and adults), which are otherwise rarely described as pollinators of angiosperms (Wardhaugh 2015). *Vincetoxicum hirundinaria*, however, is not specialized on this group of insects, and thus has a different pollination system from its congener, *V. hainanense*, which flowers at night, emits a strong, nauseating scent of rotten fruit, and relies on cockroaches for outcrossing (Xiong et al. 2020).

### Pollination effectiveness and pollination success

The proportion of pollinaria removed from flowers of *V. hirundinaria* during the day was 21%–28%, slightly below the Apocynaceae average of c. 35% (Johnson and Harder 2023). In the single population Gaisberg, where nocturnal removal occurred, the rate was higher (44%). However, PTE in the study species was very low (<11%) in comparison to other Apocynaceae (c. 25%; Johnson and Harder 2023). Still, the highest PTE was found at night (Gaisberg), which is consistent with other studies that found higher pollination effectiveness of nocturnal pollinators when compared with diurnal ones (Fleming et al. 1996, Young 2002, Vanderplanck et al. 2020). Gaisberg was the only site where pollinia were successfully inserted (and removed) at night. This may reflect the greater abundance of moths and cockroaches at Gaisberg, although their individual contributions to nocturnal pollen transfer could not be separated. In contrast, nocturnal pollinators at other sites performed no pollinaria removal or pollinia insertion, suggesting that especially night-active ants, that dominated the visitor spectra at these sites at night, do not contribute to pollination. Ants lack the pollen-transporting structures and mobility of flying insects and have been considered poor pollinators (Lubbock 1883). Nevertheless, recent studies show ant pollination occurs in some systems (Beattie 2006, de Vega et al. 2009, Schiestl and Glaser 2012), including other Apocynaceae (Domingos-Melo et al. 2017) and in *V. hainanense* (Xiong et al. 2020). Together, these findings indicate that the observed spatio-temporal variation in pollinator assemblages might be driven by differences in environmental parameters and population size among sites.

The low PTE recorded at our study sites, the finding that several insects visit multiple flowers of single plants, likely performing geitonogamy, the self-incompatibility of *V. hirundinaria*, pollen limitation at the initial fruit set stage, and substantial fruit abortion following hand cross-pollination, consistent with resource limitation during fruit maturation, overall resulted in a very low mature fruit set (2.0%). This phenomenon of low fruit set is quite common in the Asclepiadoideae subfamily (c. 1%–5%; Wyatt and Broyles 1994, Vieira and Grabalos 2003, Raju and Ramana 2009). Specifically, our results indicate that *V. hirundinaria* is self-incompatible in Salzburg, differing from Leimu (2004), in which 30%–50% of individuals were reported to be self-fertile, showing that mating-system expression varies among populations.

### Specialization and generalization in Asclepiadoideae and *Vincetoxicum*

The flower morphology of *V. hirundinaria* is highly specialized, as in all Asclepiadoideae, whereas its flower visiting and pollinating insects are particularly diverse, indicating a highly generalized pollination system in this species. The specialized pollinarium/gynostegium mechanism may therefore accommodates a surprisingly broad range of pollinator morphologies. In our study, pollinaria became attached to different body parts in different insect groups, including mouthparts, labial palps, proboscides, and legs. Thus, rather than restricting pollen transfer to a single narrowly defined pollinator morphology, the same floral mechanism can operate with several functionally distinct visitor groups. At the same time, the trapping of some visitors and the low PTE observed here show that this mechanical filtering is not uniformly effective. Morphological specialization in *V. hirundinaria* should therefore be viewed as compatible with, rather than necessarily enhancing, ecological and functional generalization.

Plants in the subfamily Asclepiadoideae all exhibit highly specialized floral structures (Frye 1902). These specialized floral traits result in varying degrees of specialization or generalization in the pollination systems of plants within this subfamily. Specialized species typically have only one or a few species of pollinators, whereas generalized species may have dozens or even over a hundred pollinator species (Ollerton et al. 2007, Armbruster 2017). For instance, members of the genus *Pachycarpus* have flowers with cryptic coloration and emit a distinctive floral scent that is highly attractive to just a few pompilid wasps. Additionally, the alkaloids present in their nectar are toxic to other pollinators, such as bees. This combination of specialized floral traits has driven the evolution of a highly specialized pollination system (Shuttleworth and Johnson 2006, 2009). On the other hand, highly generalized pollination systems have evolved within several lineages of Asclepiadoideae (Ollerton and Liede 1997). The genus *Asclepias*, for example, includes species with exceptionally diverse pollinator assemblages: more than 150 pollinators have been documented for the well-studied *A. syriaca* (Ollerton et al. 2019), while 140 potential pollinator species were recorded for *A. angustifolia* in southeastern Arizona (Behrstock 2021). More recent analyses have emphasized that specialization in Asclepiadoideae occurs at different levels: species may be functionally specialized on a single broad group of pollinators while remaining ecologically generalized through interactions with numerous species within that group. Across Apocynaceae, almost three-quarters of species with well-supported pollination data have unimodal pollination systems, but genuinely multimodal insect-generalist systems are also widespread, particularly within derived groups of Asclepiadoideae, such as *Asclepias*, Cynanchinae, and Oxypetalinae (Ollerton et al. 2019). Thus, the highly specialized floral morphology characteristic of Asclepiadoideae does not necessarily imply ecological or functional specialization. The genus *Vincetoxicum* is the third largest genus (more than 250 species) of Apocynaceae (Endress et al. 2018). Confirmed pollinators of the genus *Vincetoxicum* include flies, moths, bees, beetles, butterflies, and even cockroaches and ants (Xiong et al. 2020). The pollination system of *Vincetoxicum* plants ranges from highly specialized to highly generalized (as in *V. hirundinaria*). Historically, research on the flowering biology and pollination ecology in *Vincetoxicum* has been restricted to two temperate evolutionary groups, the Far Eastern clade and the *Vincetoxicum* s.s. clade (Liede-Schumann et al. 2016), where fly pollination predominates (Ollerton et al. 2019). Recent work on *V. sangyojarniae* has now extended this to the Asia–Africa clade (Kidyoo et al. 2024). Moth or beetle pollination was initially reported in the Far Eastern clade. Although Africa has a greater diversity of *Vincetoxicum* plants, pollination biology has only been studied in one species, *V. cernuum*, with potential pollinators including birds and beetles (Woodell 1979). Currently, only one *Vincetoxicum* species, *V. biglandulosum* (Endl.) Kuntze, is known to be pollinated by butterflies (Kato and Kawakita 2004). Unfortunately, no pollinator information is available from the Australian clade or the night-flowering and scented *V. philippicum* from Philippines. Besides *V. philippicum*, there are two other nocturnal and scented species within *Vincetoxicum*, *V. hainanense* and *V. sangyojarniae*, which are specialized for pollination by cockroaches (Xiong et al. 2020) and cecidomyiid flies (Kidyoo et al. 2024), respectively. Due to their specialized floral structures, *Vincetoxicum* plants largely rely on pollinators for successful pollination and reproduction. However, there are exceptions, a few species are capable of successful reproduction without pollinators through self-pollination mechanisms and self-compatibility, i.e. *V. hirsutum* (Wall) Kuntze (Chaturvedi 1988), *V. rossicum* (Kleopow) Barbar., *V. nigrum* (L.) Moench and *V. matsumurae* (T. Yamaz.) H. Ohashi (Yamashiro et al. 2008). Our findings indicate that *V. hirundinaria* has the most generalized pollination system documented to date within *Vincetoxicum*.

In *Vincetoxicum* and the entire Asclepiadoideae subfamily, some flower visitors are unable to remove the pollinaria from the gynostegium due to insufficient strength, then become trapped and die, others forcibly abandon body parts such as legs or mouthparts to escape but ultimately perish (Cottam 1922, Woodson 1930, Proctor and Yeo 1973, Waddington 1976, Lumer and Yost 1995, Raju et al. 2005). The most popular example should be the South American moth catcher vine, *Araujia sericifera* Brot., a widespread invader in the tropics worldwide (Stearns 1887, Coombs and Peter 2010). In this study, we observed similar but exceptionally extensive occurrences in *V. hirundinaria*. Flower visitors, primarily flies from 8 families and 16 species, became trapped while collecting nectar due to the specialized gynostegium and guiding rails of the flowers. These flies often found their mouthparts stuck in the slit of corpusculums, making it difficult to remove the pollinaria or escape. As a result, they were unsuitable as effective pollinators.

The trapping of numerous visitors raises the question of whether this interaction imposes costs on plant reproduction. Trapped insects consume nectar and may physically obstruct access to the gynostegium by subsequent effective pollinators, a possibility already considered by Knuth (1899) for *V. hirundinaria*. The very broad visitor spectrum suggests that the flowers possess traits attractive to a wide range of insects, but our study did not identify the relative contributions of floral scent, nectar, colour, or other cues to this attraction. Determining which floral traits generate this broad visitor assemblage, and whether trapped insects measurably reduce subsequent pollen transfer, would be useful subjects for future study.

## Conclusions

We for the first time investigated the pollination ecology of *Vincetoxicum hirundinaria* in Central Europe. Our findings indicate that this species attracts a large number of visitors and pollinators, with nighttime pollinators being particularly effective. We conclude that *V. hirundinaria* is morphologically highly specialized yet exhibits a highly generalized pollination system. The flowers attract an unusually broad range of insects, including species that appear poorly suited to pollination and may become trapped in the gynostegium, potentially obstructing access by effective pollinators. Future studies should test the extent to which trapped insects affect pollen transfer and identify the floral cues responsible for attracting such a diverse assemblage of visitors.

## Supplementary Material

plag048_Supplementary_Data

## Data Availability

The raw data are available as Supporting Information.
