# Supplementary material for: Generalized pollination of morphologically specialized Vincetoxicum hirundinaria (Apocynaceae, Asclepiadoideae)
Source: AoB Plants. 2026 Oct 5;18(5):plag048. doi: 10.1093/aobpla/plag048 (PMC13675644; doi:10.1093/aobpla/plag048)
Supplement: Supplementary file 1 [file plag048_supplementary_data.zip › Supplemental Data.docx]

**Supplementary information**

**Appendix S1.**

**Table S1. 1** Study sites, daytime and nighttime observation hours, and the total number of visits recorded to flowers of *Vincetoxicum hirundinaria* during the day and at night.

| Site | Elevation | Total daytime observation hours | Total nighttime observation hours | N total  day visits | N total night visits |
| --- | --- | --- | --- | --- | --- |
| Obergnigl | 560 m | 3 | 3 | 166 | 80 |
| Kapuzinerberg | 620 m | 3 | 2 | 131 | 76 |
| Kapuzinerkloster | 510 m | 3 | 2 | 216 | 54 |
| Botanical Garden | 420 m | 10 | 3 | 1307 | 103 |
| Gaisberg | 1200 m | 11 | 10 | 469 | 329 |

**Figure S1. 1** (A) *Vincetoxicum hirundinaria* and its habitat. (B) Study sites of *Vincetoxicum hirundinaria* in Salzburg and the number of flowering individuals at the different sites.

**Figure S1. 2** Floral morphology of Asclepiadoideae (Nam & Chung, 2018). (A) Flower. (B) Gynostegium with corona. (C) Gynostegium. (D) Gynoecium. (E) Pollinaria. AA, anther appendage; AN, anther; AS, anther sac; AW, anther wing; CL, corolla; CN, corona; CO, corpuscle; GR, guide rail; GY, gynostegium; OV, ovary; PE, pedicel; PO, pollinia; SC, stigmatic chamber; SH, style head; ST, style; TR, translator arm.

**Figure S1. 3** Floral morphology of *Vincetoxicum hirundinaria*. (A) Flower. (B) Gynostegium and corona. (C) Gynostegium in lateral view. (D) Pollinarium. AA, anther appendage. CL, corolla. CN, corona. CO, corpusculum. GR, guide rail. GY, gynostegium. PO, pollinium. PR, pollinaria. SC, stigmatic chamber. ST, stamen. TR, translator arm.

**Appendix S2.**

**Table S2. 1** Number of species of the different insect (super)families overall recorded on flowers of *Vincetoxicum hirundinaria*.

| Order | (Super)family | No. of species |
| --- | --- | --- |
| Blattodea | Blattellidae | 1 |
| Coleoptera | Byturidae | 1 |
|  | Cantharidae | 1 |
|  | Cerambycidae | 1 |
|  | Oedemeridae | 1 |
|  | Nitidulidae | 1 |
|  | Curculionidae | 1 |
|  | Melyridae | 1 |
|  | Scarabaeidae | 1 |
| Diptera | Anthomyiidae | 5 |
|  | Calliphoridae | 9 |
|  | Ceratopogonidae | 1 |
|  | Chloropidae | 1 |
|  | Empididae | 6 |
|  | Empidoidea | 4 |
|  | Fanniidae | 1 |
|  | Lauxaniidae | 2 |
|  | Muscidae | 1 |
|  | Oestroidea | 1 |
|  | Sarcophagidae | 1 |
|  | Scathophagidae | 1 |
|  | Sciaridae | 1 |
|  | Syrphidae | 4 |
|  | Tephritidae | 2 |
| Hemiptera | Lygaeidae | 1 |
|  | Miridae | 3 |
| Hymenoptera (ants) | Formicidae | 6 |
| Hymenoptera (bees and wasps) | Apidae | 2 |
|  | Colletidae | 1 |
|  | Halictidae | 3 |
|  | Vespidae | 1 |
| Lepidoptera | Adelidae | 1 |
|  | Crambidae | 3 |
|  | Erebidae | 1 |
|  | Geometridae | 5 |
|  | Noctuidae | 2 |
|  | Pyralidae | 1 |
|  | Pterophoridae | 1 |
| Mecoptera | Panorpidae | 1 |

**Figure S2. 1** Flower visitors of *Vincetoxicum hirundinaria* during the daytime at the different study sites. The total number (n) of insect visits recorded is also given.

**Figure S2. 2** Flower visitors of *Vincetoxicum hirundinaria* during the nighttime at the different study sites. The total number (n) of insect visits recorded is also given.

**Appendix S3.**

**Table S3. 1** (Super)family, number of species and individuals of Diptera floral visitors trapped and dying in flowers of *Vincetoxicum hirundinaria*, as well as their body and probiscis lengths (Mean ± SD) values and no. of individuals examined are given.

| Floral visitors | No. of species | No. of  individuals | Body length  (mm) | Probscis length  (mm) | No. of  Individuals  examined |
| --- | --- | --- | --- | --- | --- |
| Anthomyiidae | 2 | 4 | 10.9 ± 2.8 | 2.2 ± 0.3 | 4 |
| Ceratopogonidae | 2 | 2 | 2.7 ± 0.2 | 0.3 ± 0.1 | 2 |
| Chloropidae | 2 | 2 | 4.1 ± 0.1 | 1.1 ± 0.1 | 2 |
| Empididae | 4 | 6 | 8.5 ± 1.6 | 2.5 ± 1.0 | 4 |
| Empidoidea | 2 | 6 | 10.3 ± 5.6 | 3.6 ± 1.9 | 4 |
| Fanniidae | 1 | 2 | 5.4 ± 0.2 | 2.3 ± 0.2 | 2 |
| Oestroidea | 1 | 2 | 9.7 ± 0.5 | 2.3 ± 0.1 | 2 |
| Syrphidae | 2 | 3 | 15.6 ± 1.1 | 3.1 ± 0.2 | 3 |

**Figure S3. 1** Examples of Diptera floral visitors trapped and dying in flowers of *Vincetoxicum hirundinaria*. Scale bar = 1 mm. (A) Anthomyiidae sp., (B) Ceratopogonidae sp., (C) Chloropidae sp., (D-F) Empididae sp.,(G) Empidoidea sp., (H) Oestroidea sp., (I) Syrphidae sp..

**Table S3. 2** Number of species of the different insect families overall recorded as pollinators of *Vincetoxicum hirundinaria*.

| Order | Family | No. of species |
| --- | --- | --- |
| Blattodea | Blattellidae | 1 |
| Coleoptera | Cantharidae | 1 |
| Diptera | Anthomyiidae | 2 |
|  | Calliphoridae | 5 |
|  | Empididae | 1 |
|  | Lauxaniidae | 2 |
|  | Sarcophagidae | 1 |
|  | Syrphidae | 2 |
|  | Tephritidae | 2 |
| Hymenoptera-ants | Formicidae | 6 |
| Hymenoptera-bees and wasps | Apidae | 2 |
|  | Colletidae | 1 |
|  | Halictidae | 3 |
|  | Vespidae | 1 |
| Lepidoptera | Crambidae | 2 |
|  | Erebidae | 1 |
|  | Geometridae | 3 |
|  | Noctuidae | 1 |

**Appendix S4.**

**Figure S4. 1** Pollination rate of of *Vincetoxicum hirundinaria* during daytime and nighttime at the different study sites. (A) Pollinaria removal rate. (B) Pollinia insertion rate. n: numbers of plant individuals observed.

**Table S4. 1** Number of individuals studied, the pollen transfer efficiency (PTE) of pollinators during daytime and nighttime at the different study sites. Mean and Min-Max values are given.

| Site | N | PTE/individual (%) | |  |
| --- | --- | --- | --- | --- |
|  | daytime nighttime | daytime | nighttime | |
| Obergnigl | 40 10 | 5.4  (0.0 ~ 50.0) | – | |
| Kapuzinerkloster | 30 10 | 7.2  (0.0 ~ 50.0) | – | |
| Kapuzinerberg | 26 10 | 3.7  (0.0 ~ 37.5) | – | |
| Gaisberg | 20 20 | 8.5  (0.0 ~ 50.0) | 10.7  (0.0 ~ 33.3) | |
| Botanical Garden | 32 12 | 3.2  (0 ~ 50) | – | |

**References**

**Nam BM, Chung GY. 2018.** Taxonomic implications of floral morphology in the subfamily Asclepiadoideae (Apocynaceae s.l.) in Korea. *Korean Journal of Plant Taxonomy,* **48**: 172–184. doi: [10.11110/kjpt.2018.48.3.172](https://doi.org/10.11110/kjpt.2018.48.3.172).
